# One-year follow-up of patients with Palmer type IIC central perforation of triangular fibrocartilage complex tears treated with arthroscopic absorbable suture repair and conservative treatment

**DOI:** 10.3389/fsurg.2026.1668279

**Published:** 2026-03-09

**Authors:** Miao Wang, Haoliang Ding

**Affiliations:** 1 Department of Orthopedics, Jiading Branch of Shanghai General Hospital, Shanghai Jiaotong University School of Medicine, Jiading District Jiangqiao Hospital, Shanghai, China; 2 Department of Orthopedics, Tongren Hospital Affiliated to Shanghai Jiao Tong University School of Medicine, Shanghai, China

**Keywords:** arthroscopy, conservative treatment, palmer type IIC, sutures, triangular fibrocartilage

## Abstract

**Purpose:**

The aim of this study was to investigate and compare the clinical results of arthroscopic absorbable suture repair versus conservative treatment in the management of patients with Palmer type IIC central perforation of triangular fibrocartilage complex tears.

**Methods:**

Between September 2022 and February 2023, 50 patients with Palmer type IIC central perforation of triangular fibrocartilage complex tears at our hospital were retrospectively enrolled and included in this study, with a 1-year follow-up. Patients were classified into two groups for different treatment methods: 25 patients received conservative treatment and 25 patients received arthroscopic absorbable suture repair. Preoperative magnetic resonance imaging, intraoperative arthroscopic findings, and postoperative complications were recorded. Outcome measures were assessed using the visual analog scale (VAS) and the Modified Mayo Wrist Score (MMWS).

**Results:**

All patients completed the 1 year follow-up. In the conservative treatment group, the average VAS score was 3 ± 0.7 and the average MMWS was 83.3 ± 4.8. In the surgical group, the average VAS score was 2 ± 0.7 and the average MMWS was 85.1 ± 3.9. The two groups showed no statistically significant difference in MMWS values. On the other hand, statistically significant differences were demonstrated in VAS scores. Postoperative complications, including surgical site infection and wrist joint stiffness, were observed in four patients of the 25.

**Conclusion:**

There were no statistically significant differences in the clinical outcomes between the absorbable repair and conservative treatment groups. However, absorbable repair surgery could effectively alleviate patients’ pain symptoms.

## Graphical Abstract

**Figure:**
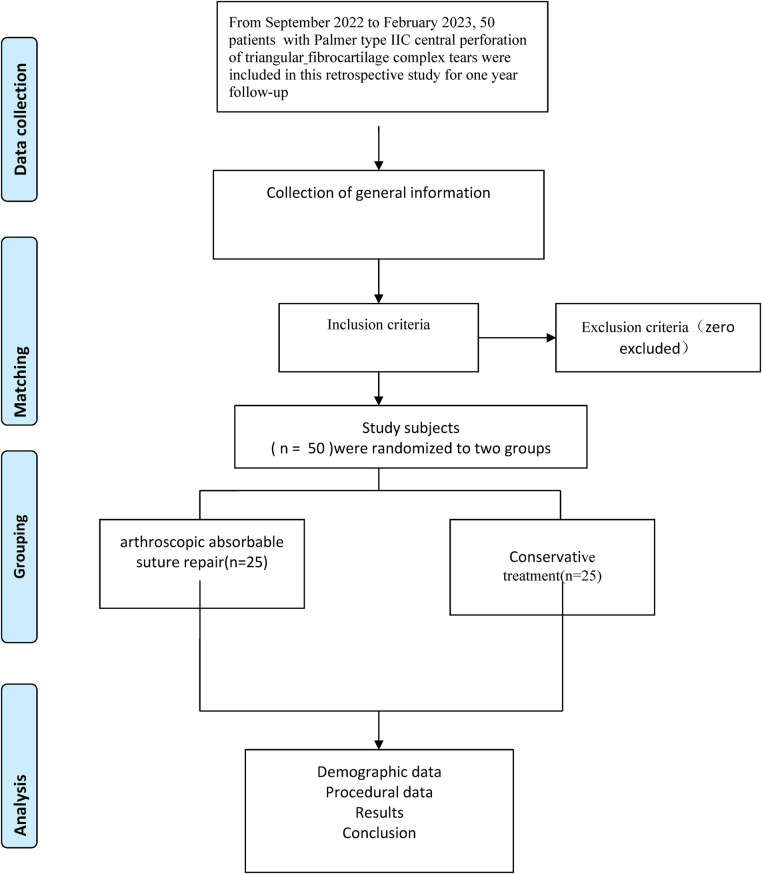

## 1 Introduction

The term “triangular fibrocartilage complex” (TFCC) was originally defined based on the distal-to-proximal perspective of the anatomical arrangement of the ligaments surrounding the wrist. These injuries can result from either traumatic events or degenerative processes, particularly among individuals who frequently overuse their wrists, such as athletes and computer programmers, due to repetitive axial loading that exceeds the TFCC's capacity. The global annual prevalence of TFCC tears in the general population is estimated to be 49% among people aged ≥70 years and 27% among people aged ≤30 years, imposing a significant burden on both affected individuals and the society (1). The TFCC is a critical stabilizing suspensory structure of the distal radioulnar joint instability (DRUJ), playing a vital role in wrist joint stabilization. However, it is often overlooked due to challenges in timely radiographic evaluation and diagnostic dilemmas in clinical practice (2, 3). Patients with TFCC pathology typically present with ulnar-sided associated wrist pain, loss of range of motion of wrist, DRUJ instability, decreased grip strength, and dysfunction, leading to varying degrees of impact on affected individuals (4). In severe cases, the sequelae following TFCC tears can lead to unemployment and difficulties in self-care. Therefore, significant emphasis should be placed on the treatment of TFCC tears, preventing progression and worsening.

The classical Palmar classification categorizes TFCC tears into IA–D and IIA–D according to the impairment location and etiology. Among the categories, type IIC tears represent a combination mechanism of wear and thinning of the TFCC, ulnocarpal impaction with lunate or ulnar chondromalacia, and chronic perforation of the triangular disk (5). Analogous to the meniscus blood supply, the peripheral region of the TFCC is well vascularized, whereas the central region is poorly vascularization, resulting in diminished healing potential (6). Therefore, significant emphasis should be placed on the theoretical treatment of type II C tears of TFCC. The visual analog scale and the Mayo Wrist Score are two common criteria for wrist function evaluation.

The treatment modalities for TFCC tears range from conservative management to open and arthroscopic transosseous techniques, as well as anchor and absorbable suture repair surgeries (7–9).

Though various treatment modalities have been employed to address TFCC tears, there remains a lack of consensus on the most effective approach (10, 11). Typically, conservative management—including immobilization, activity modification, corticosteroid injections, hand therapy, anti-inflammatory and analgesic drugs—is initially adopted for a few weeks (12). If symptoms persist and conservative management fails to provide relief, surgical treatment is proposed (13).

In recent years, the frequency of arthroscopic repair surgeries has increased due to their minimally invasive nature, although conservative treatment continues to play a crucial role. A retrospective study conducted by Spies indicated that arthroscopic debridement of type IIC central degenerative TFCC lesions is safe, reliable, and efficacious, even in cases with ulnar-positive variance (14). However, as that there is paucity of literature comparing the effectiveness of conservative treatment and arthroscopic absorbable suture repair for this type of TFCC tears, we designed this study to address this gap.

## 2 Methods

This retrospective comparative study was performed after obtaining approval from the Ethics Committee of our hospital. Patients with Palmer type IIC central perforation of triangular fibrocartilage complex tears who underwent either absorbable suture repair or conservative treatment at the hospital between September 2022 and February 2023 were included. All patients provided written informed consent. The choice of surgical repair or conservative treatment was determined by the patient's informed decision.

The group that received conservative treatment consisted of 10 men and 15 women (16 left sides and nine right sides), with an average age of 44 ± 12 years (range, 27–64 years) (Table 1). Six patients suffered from hypertension and three patients suffered from diabetes.

**Table 1:** Demographic and clinical characteristics of the patients.

Baseline characteristic	Group 1	Group 2
Number	25	25
Male	10	14
Female	15	11
Age	44 ± 12 (27–64)	43.7 ± 10.5 (28–62)

The group that received arthroscopic absorbable suture repair consisted of 14 men and 11 women (12 left sides and 13 right sides), with an average age of 43.7 ± 10.5years (range, 28–62years). Seven patients suffered from hypertension, two patients suffered from diabetes, and two patients suffered from coronary disease.

Propensity score matching was used to decrease gender difference in two groups.

### 2.1 Inclusion criterion

(1) age >18 years old; (2) Palmer type IIC central perforation of triangular fibrocartilage complex confirmed by preoperative magnetic resonance imaging; and (3) chronic course of disease longer than 3 months.

### 2.2 Exclusion criteria

(1) previous wrist surgery; (2) older than 65 years; (3) presence of with tuberculosis, infection, or tumor; and (4) history of any treatment for complex triangular fibrocartilage tears.

### 2.3 Assessment

**Figure 1:**
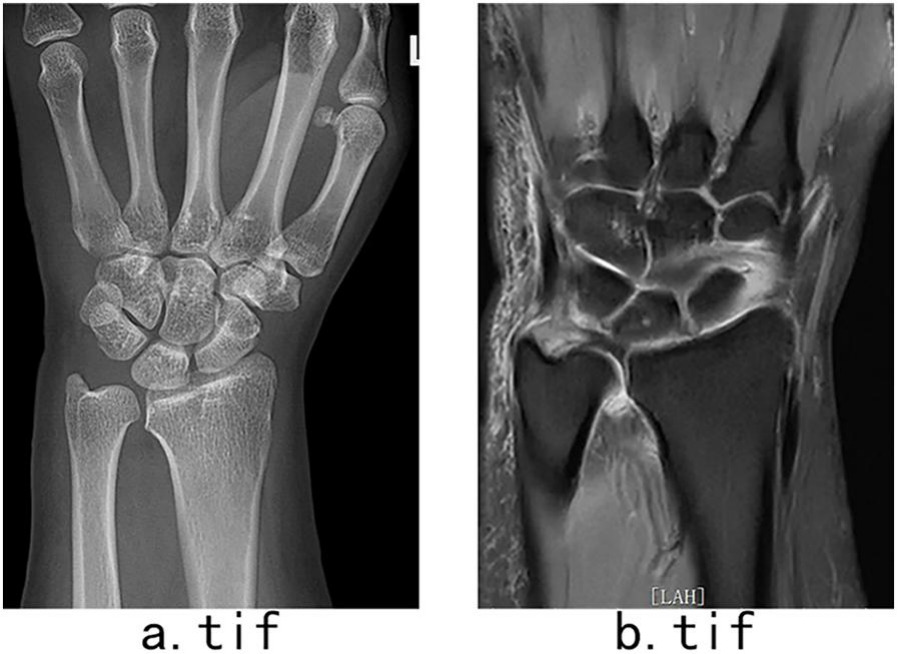
**(a)** Anterior-posterior X-ray radiograph showed no ulnar-positive variance and slight ulnar chondromalacia. **(b)** Coronal magnetic resonance radiograph showing perforation of the triangular disk.

The Modified Mayo Wrist Score (MMWS) and visual analog scale (VAS) were used to evaluate the wrist joints. Preoperative X-rays, magnetic resonance imaging findings (Figure 1), intraoperative arthroscopic pictures, and postoperative complications were recorded. A 100-mm VAS was used to assess level of pain intensity. Great levels of pain are reflected by higher numbers, and scores are sensitive to change over time and subjective perceptions.

The outcome of disability was assessed using the Modified Mayo Wrist Score Questionnaire, a validated instrument designed to evaluate patient-reported outcomes with a focus on pain, range of motion, grip strength, and ability to return to normal activities. Scores range from 0 to 100, where 0 presents the worst wrist disability and 100 indicates the best. Higher scores reflect better functional outcomes, and scores are sensitive to change over time and functional recovery. It is a reliable assessment approach.

### 2.4 Conservative treatment

Twenty-five patients in the conservative treatment group were immobilized using long-arm splinting in supination for 6 weeks, and active ROM exercises of the wrist were initiated thereafter.

In our rehabilitation protocol, isometric extensor carpi ulnaris and pronator quadratus was allowed in first 6 weeks after the surgery.

Subsequent training without splint started with activating muscles, progress weight bearing training, 2–3 min ×3 sets ×4/week.

### 2.5 Surgical procedure

All surgeries were performed under general anesthesia by an experienced surgeon with the use of a tourniquet, keeping the patient in supine position.

**Figure 2:**
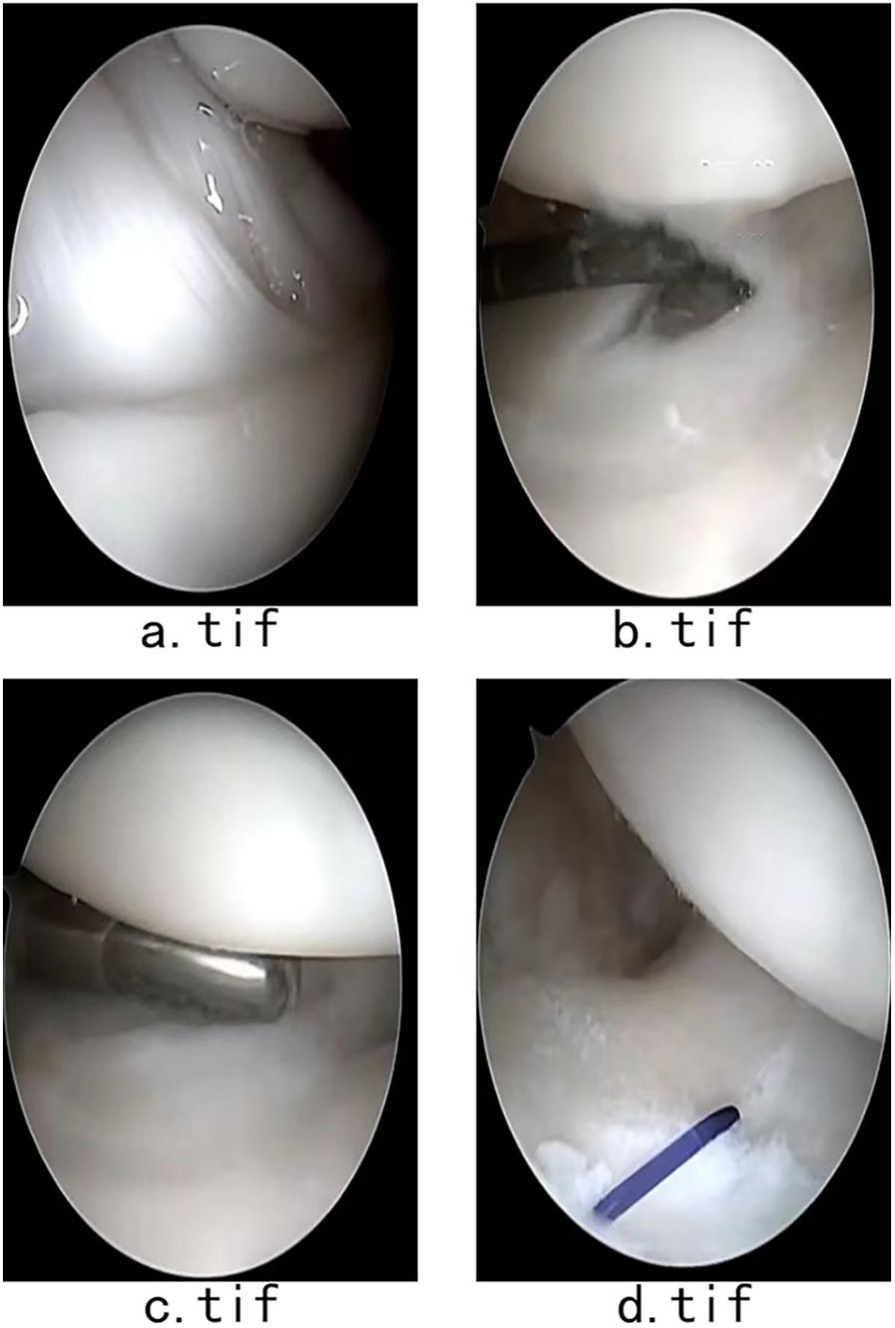
**(a)** Arthroscopic examination of the radiocarpal joint, surrounding ligaments, and bone. **(b)** The Palmer 2C type of triangular fibrocartilage complex tears was diagnosed through the visualization of perforation of the triangular disk. **(c)** TFCC and associated synovitis debrided. **(d)** Tensioning performed after the ruptured triangular disk was fully repaired.

Routine arthroscopy incisions were made following arthroscopic examination of the radiocarpal joint, surrounding ligaments, bone, and central triangular disk. Palmer 2C type triangular fibrocartilage complex tears were diagnosed through visualization of the perforation of the triangular disk. Following the completion of diagnostic arthroscopy, the TFCC and associated synovitis were debrided. Arthroscopic TFCC repair was then carried out using the outside-in technique with a 20G needle loaded with 3-0 absorbable suture (Johnson & Johnson, New Brunswick, NY, USA), penetrating the articular disk radial to the edge of the tear. Prior to tensioning, complete repair of the ruptured triangular disk was confirmed (Figure 2). Postoperatively, a long-arm splint was applied for 6 weeks, and active ROM of the wrist was initiated 6 weeks after surgery.

### 2.6 Follow-up

Both groups were evaluated at 1, 3, 6 months, and 1 year.

## 3 Results

All patients in both the conservative treatment group (10 male and 15 females) and arthroscopic surgery group (14 males and 11 females) completed postoperative 1-year follow up. At 1 year follow-up, MMWS values increased from 60.5 ± 7.4 to 83.3 ± 4.8 in the conservative group and from 60.7 ± 5.2 to 85.1 ± 3.9 in surgical group, and VAS scores decreased from 6.6 ± 1.1 to 3 ± 0.7 in the conservative group and from 6.3 ± 1.1 to 2 ± 0.7 in the surgical group (Tables 2, 3). The two groups showed no statistically significant differences in MMWS values. A statistically significant difference was observed in VAS scores (Table 4).

**Table 2:** VAS and MMWS in conservative treatment.

Scoring system	Pretreatment	After treatment	*t*
VAS	6.6 ± 1.1	3 ± 0.7	18.8
MMWS	60.5 ± 7.4	83.3 ± 4.8	−26.149

**Table 3:** VAS and MMWS in arthroscopic absorbable suture repair treatment.

Scoring system	Preoperative	Postoperative	*t*
VAS	6.3 ± 1.1	2 ± 0.7	16.438
MMWS	60.7 ± 5.2	85.1 ± 3.9	−21.886

**Table 4:** Comparison of clinical outcomes between the two groups.

Scoring system	Conservative group	Repair group	*p*
VAS	3 ± 0.7	2 ± 0.7	0.001
MMWS	83.3 ± 4.8	85.1 ± 3.9	0.198

Four surgery-related complications were observed, including one surgical site infection and three cases of wrist stiffness. No major complications were reported.

### 3.1 Statistical analysis

The Wilcoxon rank-sum test and paired *t*-test were used for measurement and counting data. A *P*-value <0.05 was considered statistically significant. All statistical analyses were performed using SPSS Statistics version 25.0 software (IBM Corp., Armonk, NY, USA).

## 4 Discussion

In this study, there were no statistically significant differences in clinical outcomes between the absorbable repair and conservative treatment groups, although absorbable repair surgery effectively alleviated patients' pain symptoms.

Significant bodies of literature have addressed various types of TFCC tears. Claire's cadaver study suggested that transosseous tunnel repair might be a good technique for type IB TFCC tears (13). Lenartowicz reported that chronic Palmer type 1C TFCC injuries can be successfully treated with extensor carpi ulnaris reconstruction of the ulnotriquetral ligament (15). A study conducted by Spies concluded that arthroscopic debridement of central degenerative type IIC TFCC lesions is safe, reliable, and efficacious, even in patients with ulnar-positive variance (14). Related research indicates that surgery is not superior to conservative management in patients with degenerative meniscal lesions and poorly vascularized segments (16).

In our study, a total of 50 patients were divided into two groups to assess and compare the clinical results of arthroscopic absorbable suture repair or conservative treatment. Postoperative VAS and MMWS values improved statistically in both groups, demonstrating that both treatment modalities achieve satisfactory clinical outcomes and relieve pain. Comparison of 1-year follow-up outcomes showed no statistically significant difference in MMWS values, whereas a significant difference was observed in VAS scores, indicating that absorbable repair surgery can better alleviate patients’ pain symptoms.

Arthroscopy is considered the gold standard for diagnosing TFCC tears (17). Despite the potential benefits of arthroscopic surgery and magnetic resonance arthrography, their widespread application is limited due to invasiveness, procedural duration, and high costs. In addition, success is highly operator-dependent. MRI remains crucial for diagnosis, but the TFCC's small and thin anatomical structure often leads to diagnostic challenges.

Various arthroscopic techniques (inside-out, outside-in, all-inside) and surgical procedures (open or arthroscopic repair, arthroscopic debridement, ulnar shortening, Wafer procedure) have been reported. Debridement trims the tear edge to enhance healing by improving blood supply. While debridement benefits central TFCC tears, it is associated with poorer outcomes in type II tears with positive ulnar variance (18).

Overall, these techniques have been reported with good-to-excellent results in the literature (19–21). A retrospective study conducted by Yousef Khair showed that open repair of TFCC tears using either microanchor or transosseous techniques led to pain-free range of motion, improved grip strength, and stable DRUJ, with no significant differences in clinical outcome between the two techniques (1).

Arthroscopic-assisted transosseous suture anchor repair has proven successful with minimal invasiveness, without requiring open capsulotomy. In our study, absorbable sutures facilitated a simpler procedure compared to suture anchor repair, resulting in significant pain relief and cost-effectiveness. Overall, the literature reports good-to-excellent outcomes with these techniques (19–21). Retrospective studies by Yousef Khair and Chia Hung Hung reported comparable improvements in pain-free range of motion, grip strength, and DRUJ stability with various TFCC repair techniques (1) Chia Hung Hung's retrospective study revealed that both the all-inside arthroscopic suture anchor technique and the arthroscopic transosseous suture technique are appropriate treatments to treat TFCC tears.

The TFCC is anatomically complex, consisting of dorsal and palmar ligaments spanning radius and ulna, ulna-carpal ligaments, a meniscal homolog, and the subs heath of the ulnar wrist extensor. Its biomechanics render it susceptible to injury, particularly the articular disk, which is prone to radial injury due to intersecting radial-oriented fibers with central oblique fibers.

Postoperative complications include nerve injury, extensor tendon injury and tendinitis, fracture, stiffness, and persistence of symptoms. Attention to anatomical detail and careful manipulation help reduce complications that could lead to ongoing pain and dysfunction (22).

## 6 Strengthens and limitations

Similar to meniscus injuries, type IIC TFCC tears with perforation of the triangular disk have reduced healing potential due to poor vascular supply. To our knowledge, no prior research has compared arthroscopic absorbable suture repair and conservative treatment for Palmer type IIC central perforation of TFCC tears. Therefore, this study was designed to investigate and compare the clinical results of these two treatment modalities.

This study has several limitations: (1) The sample size was relatively small and the study was retrospective. (2) Only a limited number of measures were used to evaluate clinical outcomes. To settle this problem, future research should conduct prospective studies with longer follow-up periods and large size samples.

## Data Availability

The original contributions presented in the study are included in the article/Supplementary Material, further inquiries can be directed to the corresponding author/s.
